# A Hybrid GRA-TOPSIS-RFR Optimization Approach for Minimizing Burrs in Micro-Milling of Ti-6Al-4V Alloys

**DOI:** 10.3390/mi16040464

**Published:** 2025-04-14

**Authors:** Rongkai Tan, Abhilash Puthanveettil Madathil, Qi Liu, Jian Cheng, Fengtao Lin

**Affiliations:** 1 School of Mechatronics and Vehicle Engineering, East China Jiaotong University, Nanchang 330013, China; tanrongkai17@163.com (R.T.); menggu_lin@163.com (F.L.); 2 Centre for Precision Manufacturing, DMEM, University of Strathclyde, Glasgow G1 1XJ, UK; 3 National Manufacturing Institute Scotland (NMIS), Renfrew PA3 2EF, UK; 4 Department of Mechanical Engineering, University of Bath, Bath BA2 7HA, UK; 5 State Key Laboratory of Robotics and System, Harbin Institute of Technology, Harbin 150001, China; cheng.826@hit.edu.cn

**Keywords:** Ti6Al4V alloys, micro-milling, parameter optimization, Taguchi method, grey relational analysis, random forest, GRA-TOPSIS

## Abstract

Micro-milling is increasingly recognized as a crucial technique for machining intricate and miniature 3D aerospace components, particularly those fabricated from difficult-to-cut Ti-6Al-4V alloys. However, its practical applications are hindered by significant challenges, particularly the unavoidable generation of burrs, which complicate subsequent finishing processes and adversely affect overall part quality. To optimize the burr formation in the micro-milling of Ti-6Al-4V alloys, this study proposes a novel hybrid-ranking optimization algorithm that integrates Grey Relational Analysis (GRA) with the Technique for Order Preference by Similarity to Ideal Solution (TOPSIS). This approach innovatively combines GRA and TOPSIS with a random forest regression (RFR) model, facilitating the exploration of nonlinear and complex relationships between input parameters and machining outcomes. Specifically, the effects of spindle speed, depth of cut, and feed rate per tooth on surface roughness and burr width generated during both down-milling and up-milling processes were systematically investigated using the proposed methodology. The results reveal that the depth of cut is the most influential factor affecting surface roughness, while feed rate per tooth plays a critical role in controlling burr formation. Moreover, the GRA-TOPSIS-RFR method significantly outperforms existing optimization and prediction models, with the integration of the RFR model enhancing prediction accuracy by 42.6% compared to traditional linear regression approaches. The validation experimental results agree well with the GRA-TOPSIS-RFR-optimized outcomes. This research provides valuable insights into optimizing the micro-milling process of titanium components, ultimately contributing to improved quality, performance, and service life across various aerospace applications.

## 1. Introduction

Titanium alloys are widely used in aerospace, petrochemical, and automobile fields due to their high strength, good corrosion resistance, and high heat resistance [1,2]. Recently, the products made from titanium alloys in these fields have been further developed towards miniaturization with their functional structural features in micrometres [3,4]. These miniaturized products normally have complex three-dimensional geometric structures and require high machining quality, creating a great demand for the development of micro-milling technology [5] as this method is powerful for producing sub-micron machining accuracy in machining freeform surface structures [6]. However, titanium alloys, especially the Ti-6Al-4V alloy, are known as one typical difficult-to-machine material [7,8] due to their poor machinability and the difficulty in the mechanical cutting process [9,10]. For example, the presence of burrs is unavoidable in the micro-milling processes of titanium alloys [11], which not only reduces the quality and accuracy of the machined surface but also causes the wear of milling cutters [12]. As a result, it affects the processing efficiency and increases production costs. The traditional deburring methods (e.g., manual deburring and mechanical deburring) in macro-milling processes cannot be directly applied to remove micro-scale burrs in micro-milling since these methods may not be appropriate to trim the smaller and complex geometrical structures on the processed miniaturized products [13,14]. Hence, there is a pressing need for in-depth investigations into the optimization of burrs during micro-milling processes of Ti-6Al-4V alloys. Such exploration is crucial to improve the machined surface quality and further enhance their service performance and lifespan.

Considerable efforts have been dedicated to investigating the formation mechanism of milling burrs in titanium alloy processing [15,16]. Numerous three-dimensional finite element models (FEMs) have been constructed to simulate the micro-milling process of the Ti-6Al-4V titanium alloy [17,18]. These models have provided insights into the impact of tool geometry, blunt circle radius of the tool cutting edge, as well as the coating, on the process of burr formation and the resulting burr sizes. Through FEM simulation and experimental analysis, Chen [11] systematically classified the types of burrs occurring in the microgroove milling process of Ti-6Al-4V and reported that the size of milling burrs generated on the up-milling side is much larger than that on the down-milling side. Recently, with the development of digital-driven artificial intelligence (AI) technologies [19,20], a lot of digital-driven analysis methods have been adopted to optimize the machining surface quality and reduce the burr sizes, like multi-objective optimization, grey correlation analysis (GRA), and particle swarm algorithm [15,21]. These methods have their respective pros and cons. Meanwhile, hybrid AI approaches, integrating some of the abovementioned methods are gradually showing their powerful capacities to reveal the relationship between processing factors and machining responses [22,23].

Several hybrid approaches have been proposed in the past to enhance the performance of multi-criteria decision-making (MCDM) processes like GRA. Dhuria [24] developed a hybrid GRA approach with entropy weight assignment during ultrasonic machining of the Ti-6Al-4V alloy. The entropy weight method assigns more weights to the response with higher variation and is regarded as a better approach than uniform weights. The GRA-PCA approach was used to study the radial overcut of D2 steel during EDM processing by Pradhan [25]. Though the parametric study and optimization provided better overall performance, the R2 value for the GRG model was found to be just 0.83. The GRA PCA approach was also used by Kharwar and Verma [26] during the drilling of carbon nanocomposites and by Umamaheswarrao [27] during the hard turning of AISI steels. With the objective of optimizing abrasive water jet machining, a grey-SVM approach was introduced by Deris [28]. The advantage of this approach with respect to other hybrid methods is its ability to suppress the redundant features from the dataset. Dewangan [29] used a hybrid-grey fuzzy approach for the multi-objective optimization of the EDM process to affect a 10% overall improvement compared to the initial condition.

Similarly to optimization, several approaches are explored in recent years for machining response prediction. Among those, machine learning (ML)-based approaches are reported to be the best performing in terms of prediction accuracy. A few of the latest advancements in ML-based machining response prediction are discussed henceforth. Rajamani [30] has used an adaptive neuro-fuzzy inference system (ANFIS) model and whale optimization algorithm (WOA) to predict and optimize process responses during the laser machining of Hastelloy C276. The responses considered were cutting rate, kerf taper, and surface finish. The results are very promising with an R^2^ value of 97% for prediction and an enhanced performance upon optimization. ANFIS model-based response prediction was also attempted by Manikandan [31] for the wire EDM processing of MMCs. The drilling process was modelled by Latha and Senthilkumar [32] using a fuzzy logic model. The criticism of the fuzzy model is its lack of flexibility and over-dependence on expert knowledge for the rule set creation. For a process with a material removal mechanism that is not completely understood, creating a fuzzy-based system can be tedious and inaccurate. Together with FEM analysis, a random forest regression (RFR) model was developed for microhardness prediction by Arisoy and Özel [33] during the turning operation of Ti-6Al-4V. Prashanth et al. [34] compared SVM, Gaussian regression, and ensemble techniques for grinding response prediction under different cooling environments. Curiously, the Gaussian regression method was found to outperform the other two in terms of R^2^ and RMSE performance matrices.

From the literature, it was understood that there is an active scope for the optimization and response prediction of milling parameters through advanced computational and statistical techniques. Though the GRA method has the potential to select the optimal machining conditions, performance enhancement is often very marginal. Hybrid techniques which combine the advantages of multiple MCDM methods can be looked into to resolve this limitation. Additionally, the literature review highlighted that ML prediction models like fuzzy logic rely on operator knowledge to set the rules and are not trainable. From an industrial production context, maximizing product quality measures are very critical. A comprehensive investigation of the influence of machining parameters on micro-milling is thus performed towards processing the components that comply with industrial requirements. Therefore, this paper aims to perform micro-groove milling experiments in titanium alloys to reveal the influence of micro-milling process parameters (spindle speed, depth of cut, and feed rate) on the formation mechanism of burrs on the down-milling side and up-milling side. Additionally, a hybrid GRA-TOPSIS model is proposed in this study, based on the Taguchi experimental design. By utilizing this model, a combination of cutting process parameters that can achieve minimum machining surface roughness and burr size in the micro-milling of titanium alloy micro-miniature components is obtained. This provides valuable parameter guidance for the optimization of the micro-milling process. Furthermore, the research explores the integrating random forest regression (RFR) model to the proposed GRA-TOPSIS, which exhibits superior prediction accuracy compared to existing machine learning-based models for micro-milling applications. The introduction of the RFR model holds great potential for enhancing the accuracy of response prediction in micro-milling processes.

## 2. Experimental Design and Analysis Methods

### 2.1. Materials and Machining Set-Up

**Figure 1:**
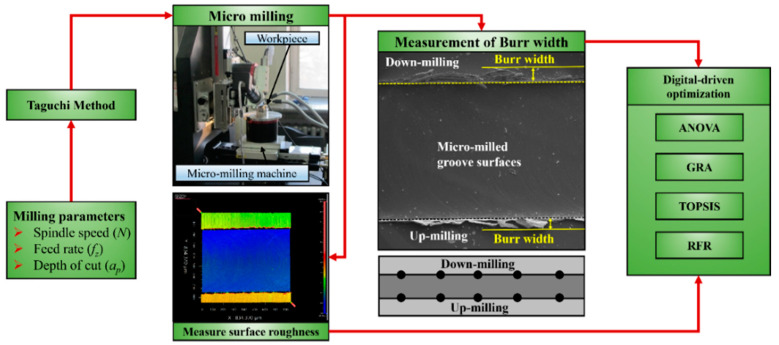
Machining set-up and the measurement schematic of burr width.

The micro-milling groove experiments were carried out on a house-built five-axis precision machine tool, which adopts a high-speed motorized spindle with a maximum speed of 80,000 rpm and also a precise three-dimensional transportation platform. A precision optical microscope was used for tool setting. Ti-6Al-4V alloys in the size of 20 mm × 20 mm × 10 mm were used for workpieces. The specific chemical composition and mechanical properties of Ti-6Al-4V alloys are listed in Table 1 and Table 2, respectively. Regarding the micro-milling grooves, their length was set as 2 mm, and the distance between adjacent grooves was 1 mm. A commercial micro-milling cutter with AlTiN coating was adopted in this experiment (HTS, Hetaisheng Tools, Shenzhen, China). This mill has a double edge, and its diameter and chamber length are 0.6 mm and 0.05 mm, respectively. The helix angle of the milling cutter is 30°. The radius of the cutting edge is about 1.631 μm. All micro-groove experiments were performed under dry-cutting conditions. There are two types of milling modes engaged in the microgroove milling tests: up-milling mode and down-milling mode [11,35], as shown in Figure 1. The division of these two types of milling modes is highly related to the change in undeformed cutting thickness (UCT) during the material removal process in one rotation of the cutting edge [36]. It can be summarized that the UCT increases from zero to the value of *f_z_* in the up-milling mode, while it decreases from the value *f_z_* to zero with the rotation of cutting edges.

After micro-milling, the workpiece was cleaned with ultrasonic waves, and the surface roughness of the micro-grooves was measured with a white light interferometer with a resolution of 0.1 nm. The micro-groove topography features, especially the burrs on the down-milling side and the up-milling side of grooves, can be observed with a scanning electron microscope (SEM). Meanwhile, the size of burrs, i.e., its width, can be measured by analyzing the obtained SEM images, as shown in Figure 1. The width values were evaluated as the average of measurement results from five different locations on the micro-milled groove surfaces.

### 2.2. Experimental Design

The micro-groove milling test was designed by using the Taguchi method. The control factors were the spindle rotation speed “*N*”, feed per tooth “*f_z_*”, and depth of cut “*a*_p_”, respectively. Based on the practical engineering practice, the spindle rotation speed “*N*” was set as 5000, 15,000, and 25,000 rev/min, and the corresponding cutting velocity is 9.43 m/min, 28.25 m/min, and 47.13 m/min; the feed per tooth “*f_z_*” was set as 0.1, 0.3, and 0.5 mm/tooth; while the depth of cut “*a_p_*” was set as 0.05, 0.15, and 0.25 mm. The specific milling parameters can be found in Table 3. After micro-groove milling tests, the proposed GRA-TOPSIS ranking was utilized in Python 3.10 to evaluate the influence of these machining parameters on the burr generation behaviours and also the machined surface qualities.

Meanwhile, the array of L_27_ types was used in this micro-groove milling test, as shown in Table 4, which meets the requirements of the Taguchi method: the total freedom degree cannot be smaller than the number of variables.

## 3. Analysis Methods for Optimizing the Milling Parameters

### 3.1. GRA-TOPSIS Method

In the past, the TOPSIS method has been widely used for multi-criteria decision-making, especially in manufacturing applications. Though the approach is easy to apply and understand, traditional TOPSIS has several fundamental shortcomings like ranking reversal [37], inability to understand relative importance based on distance from multiple reference points [38], and high subjectivity. The GRA-TOPSIS methodology is an improvement over both GRA and TOPSIS methods for multi-objective optimization. It replaces the relative geometric distance between the alternatives with the grey relational coefficient (GRC) from the Grey Relational Analysis (GRA). The method has the following steps.

(1) A decision matrix $D_{m}$ is developed considering all the responses and their alternatives. $x_{ij}$ gives the $j^{th}$ response of $i^{th}$ alternative; also, m and n are the number of alternatives and responses, respectively.
  (1) $$D_{m}=\left[\begin{array}{cccc} x_{11} & x_{12} & \ldots & x_{1n}\\ x_{21} & x_{22} & \ldots & x_{2n}\\ : & : & \ldots & :\\ x_{m1} & x_{m2} & \ldots & x_{mn} \end{array}\right]$$
(2) The normalization of the decision matrix with the following formula:
  (2) $$r_{ij}=\frac{x_{ij}}{\sqrt{\sum_{i=1}^{m}x_{ij}^{2}}}\;\;\;\;\;\;where\;j=1,2\ldots ,\;n$$
(3) The computation of the weighted normal decision matrix by choosing appropriate response weights. Weights are chosen based on relative importance. In the case of equal response significance, each weight $w_{j}=1/n$, as shown in the following:
  (3) $$v_{ij}=w_{j}r_{ij}\;,\;\;\;Given\;\sum_{j=1}^{n}w_{j}=1$$
(4) The selection of best and worst solution candidates among the weighed normalized matrix. The best value for a “lower is better” solution like surface roughness is the lowest value among the weighted normal responses and vice versa. The equations for best and worst solutions are as follows:
  (4) $$V^{+}=\left\{\left(\sum_{i}^{max}v_{ij}|j\epsilon J\right)\;,\;\left(\sum_{i}^{min}ǀ\;j\;\int J|i=1,2,\ldots \ldots ,\;m\;\right)\right\}\;=v_{1}^{+}{,\;v}_{2}^{+}v_{3}^{+}{,\ldots \ldots ,v}_{n}^{+}$$
  (5) $$V^{-}=\left\{\left(\sum_{i}^{min}v_{ij}|j\epsilon J\right)\;,\;\left(\sum_{i}^{max}ǀ\;j\;\int J|i=1,2,\ldots \ldots ,\;m\;\right)\right\}\;=v_{1}^{-}{,\;v}_{2}^{-}{,v}_{3}^{-}{,\ldots \ldots ,v}_{n}^{-}$$
(5) The positive grey relational coefficients with respect to the “best solution” are computed as
  (6) $${GRC}_{ij}^{+}=\frac{\Delta {min}_{j}+\varepsilon \Delta max}{(v_{j}^{+}-v_{ij})+\varepsilon \Delta max}$$

Similarly, the negative grey relational coefficients with respect to the “worst solution” are computed as

(7) $${GRC}_{ij}^{-}=\frac{\Delta {min}_{j}+\varepsilon \Delta max}{(v_{j}^{-}-v_{ij})+\varepsilon \Delta max}$$

where ${\Delta max}_{j}=v_{j}^{+}-v_{j}^{-}$; ${\Delta min}_{j}=v_{j}^{-}-v_{j}^{+}$; *ε* is called a distinguished coefficient with a value that is between 0 and 1.

(6) Positive and negative grey relational grades are computed as average positive and negative grey relational coefficients, respectively.
  (8) $${GRG}_{i}^{+}=\frac{\sum_{j=0}^{n}{GRC}_{ij}^{+}}{n}$$
  (9) $${GRG}_{i}^{-}=\frac{\sum_{j=0}^{n}{GRC}_{ij}^{-}}{n}$$
(7) Calculating an alternative’s relative closeness compared to the ideal solution is given by
  (10) $$P_{i}=\frac{{GRG}_{i}^{+}}{{GRG}_{i}^{+}+{GRG}_{i}^{-}}\;,\;\;i=1,2,\ldots \ldots ,m\;$$
(8) Ranking the alternatives based on the descending order of *P_i_*.

### 3.2. Random Forest Regression (RFR) Methods

The ensemble is an approach in AI that combines multiple ML models in a way to obtain better accuracy than the constituent models. Three dominant categories of ensemble techniques are stacking, bagging, and boosting. Bagging stands for bootstrap aggregation. Bagging trains several weak ML models like decision trees parallelly and use those models’ combined predictions to come up with the final output. For a regression problem, the bagged model prediction is the mean of all weak learners’ predictions. Similarly, for a classification problem, the final predicted class is the best-voted class among all the weak learner predictions.

Random forest regression is a special type of bagging which improves upon the predictive performance of conventional bagging models. Unlike earlier bagging techniques, the RFR model restricts the features to consider at any split point, in order to minimize the correlations across the data samples. This number of features “m” is taken as √p as a rule of thumb, if p is the number of model parameters. Compared to other ensemble techniques, RFR has the advantages of diversity, reduced dimensions per weak learner model, and computing optimization through parallel processing. The important hyperparameters are the number of decision trees, the number of features at the split node, and the minimum number of leaves to consider splitting. The steps involved in RFR modelling are as follows:

(1) In RFR, the “*n*” number of random data subsets are generated from the original dataset with replacement.
(2) Next, “*n*” decision tree models are trained by considering each generated data sample.
(3) The individual decision tree model predictions are recorded.
(4) The final prediction is the mean of all individual model predictions.

**Figure 2:**
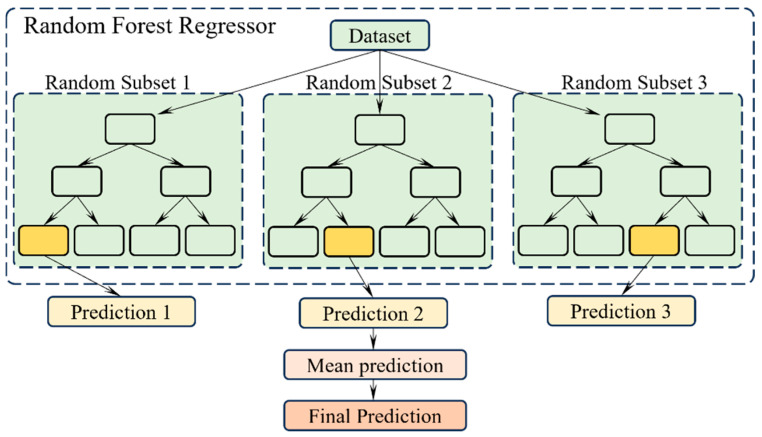
Random forest regression architecture.

A generic architecture of an RFR model is given in Figure 2.

## 4. Results and Discussion

### 4.1. ANOVA

#### 4.1.1. ANOVA of the Surface Roughness

**Figure 3:**
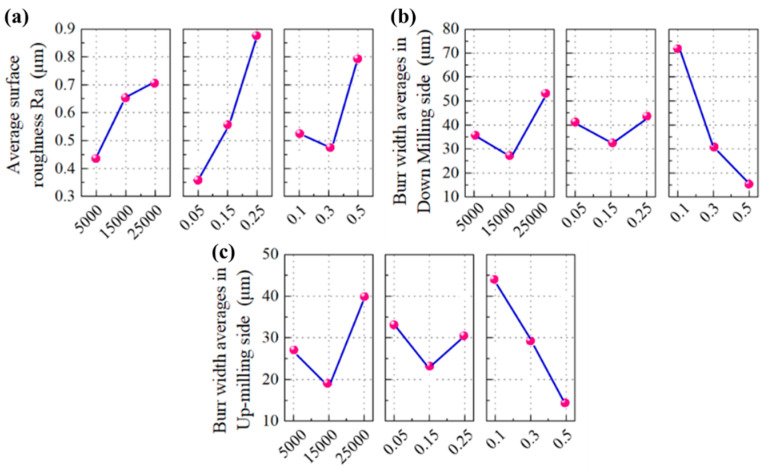
Main effect plot of ANOVA for (**a**) *R*_a_; (**b**) burr width generated in down-milling mode; (**c**) burr width generated in up-milling mode.

The measured surface roughness at different milling conditions can be found in Table 4. In order to optimize the machined surface quality, the main effect based on ANOVA was carried out to reveal the specific influence of the controlling factors, e.g., spindle speed (*N*), depth of cut (*a*_p_), and feed rate per tooth (*f*_z_), on the measured surface roughness *R*_a_, as shown in Figure 3a. It can be seen that with the rise in spindle speed, the *R*_a_ values also increase, and the minimum value of *R*_a_ was obtained at *N* = 5000 RPM. This phenomenon could be attributed to tool wear which predominantly occurs in the high-speed milling of the titanium alloy, resulting in a severe ploughing effect in the material removal process. The depth of cut has a similar effect on *R*_a_ to the spindle speed. The minimum *R*_a_ was obtained at *a*_p_ = 0.05 mm. But, when it comes to the feed rate, the change in *R*a does not remain proportional to the increase in *f*_z_. The minimum *R*_a_ was obtained at *f*_z_ = 0.03 mm/z. This is because, in the micro-milling process, if the feed rate per tooth is quite small, it could make the practical UCT less than the critical value of generating an effective shear removal of the material. Thus, *f*_z_ = 0.03 mm/z could be approximated as a critical value in this cutting test.

Meanwhile, the ANOVA results with a confidence interval of 95% are given in Table 5. One can see that the depth of cut (represented by factor B) has the largest effect on the machined surface roughness, followed by feed rate per tooth (factor C) and spindle speed (factor A). The contribution percentage of these three factors is calculated as 37.8% (B), 16.6% (B), and 11.1% (A), respectively. It is worth noting that the interaction between spindle speed and feed rate per tooth (A*C) also has an important effect on the *R*_a_ with a contribution percentage of 13.48%. However, the effect of the interaction A∗B and B∗C on *R*_a_ could be ignored as obtained *p* is higher than 0.05.

#### 4.1.2. ANOVA of the Burr Width in Down-Milling

Figure 3b shows the main effect based on ANOVA for revealing the specific influence of the controlling factors on the burr width generated in down-milling mode (WdB). It can be seen that the WdB decreases significantly with the rise in feed rate (*f*_z_). This is because the change in this factor can increase UCT, meaning the ploughing effect could play a more important role in the micro-milling process. In particular, the ploughing effect becomes dominant when the cutting edges enter and exit the samples, causing the pile-up of burrs at the groove edges. Meanwhile, the minimum WdB was achieved at a spindle speed of 15,000 RPM. The increase in WdB at high spindle speeds (*N* = 25,000 RPM) might be attributed to the tool wear. In addition, it was observed that the middle depth of cut (*a*_p_ = 0.15 mm) could contribute to the achievement of a minimum of WdB.

Table 6 presents the ANOVA analysis result for the burr width generated in the down-milling mode. It was found that the feed rate per tooth (factor C) has the largest effect on the WdB, followed by spindle speed (factor A). The contribution percentage of these two factors is calculated as 39.5% (C) and 9.0% (A), respectively. The effect of the depth of cut (represented by factor B) on WdB can be neglected as *p* > 0.05. Regarding the interactions between parameters, the B*C has an important effect on WdB with a contribution percentage of 16.3%. There is no obvious influence of B*C on WdB.

#### 4.1.3. ANOVA of the Burr Width in Up-Milling

As mentioned above, the burr widths generated in the up-milling mode (WuB) are generally smaller than WdB in the down-milling mode. One possible reason might be that, although the burr formation in up-milling has a similar mechanism to that in down-milling, e.g., the ploughing effect, the formed burrs tend to move into the groove area with the rotation of the cutter and could be removed by the next cutting processes. The main effect based on ANOVA for WuB is shown Figure 3c, which is quite similar to that in Figure 3b for WdB.

The ANOVA results for the burr width generated in the up-milling mode are presented in Table 7. It can be seen that the feed rate per tooth (factor C) exerts the most important role in WuB, having a contribution percentage of up to 43.4%. The spindle speed (factor A) and depth of cut (factor B) account for 7.2% and 8.6% of the contribution percentage on WuB. Regarding the interactions between parameters, A*B and A*C play important effects on WuB, with contribution percentages of 12.7% and 15.2, respectively. There is no obvious effect of B*C on WuB.

### 4.2. Machine Learning-Based Response Prediction

**Figure 4:**
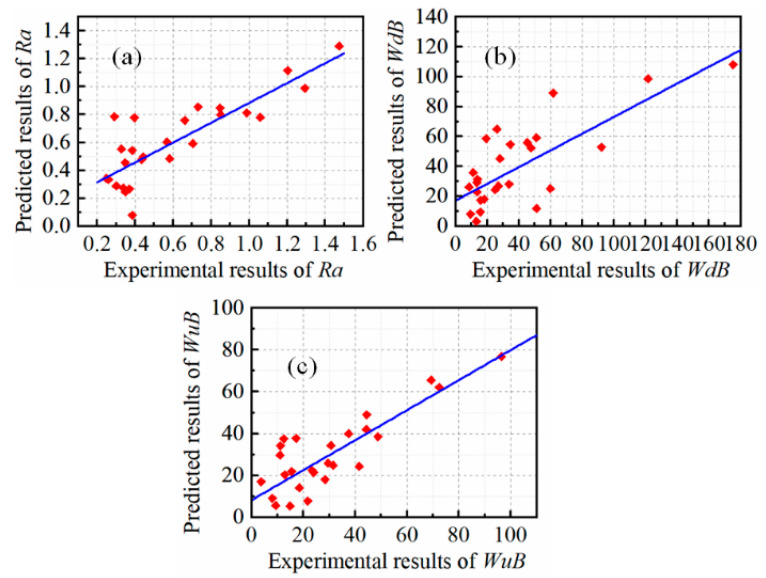
The relationship between the experiment results and estimated results: (**a**) R_a_; (**b**) WdB; (**c**) WuB.

Initially, linear regression models were built for predicting the surface roughness (*R*_a_), burr widths on the down-milling side (WdB), and burr widths on the up-milling side (WuB), as expressed in Equations (10)–(12). In addition, the R^2^ value for these three models is 0.7009, 0.5591, and 0.7164, respectively. Figure 4 shows the concordance between the experimental data, as listed in Table 2, and the predicted data by the proposed models (Equations (10)–(12)).

(11) $$\begin{array}{ll} Ra & =0.535+0.200A+0.072B-0.831C-0.0774A^{2}\\ & \;\;+0.1208A*C+0.0461B^{2}+0.1809C^{2} \end{array}$$

(12) $$\begin{array}{ll} WdB & =131.1-46.6A-53.4C+18.6A^{2}\\ & +12.8C^{2}+3.15A*B-12.83A*C \end{array}$$

(13) $$\begin{array}{ll} WuB & =104.2-38.1A+9.9C-51.2B+13.79A^{2\;}\\ & +9.09B^{2}-0.41C^{2}+6.84A*B\\ & -12.16A*C \end{array}$$

**Figure 5:**
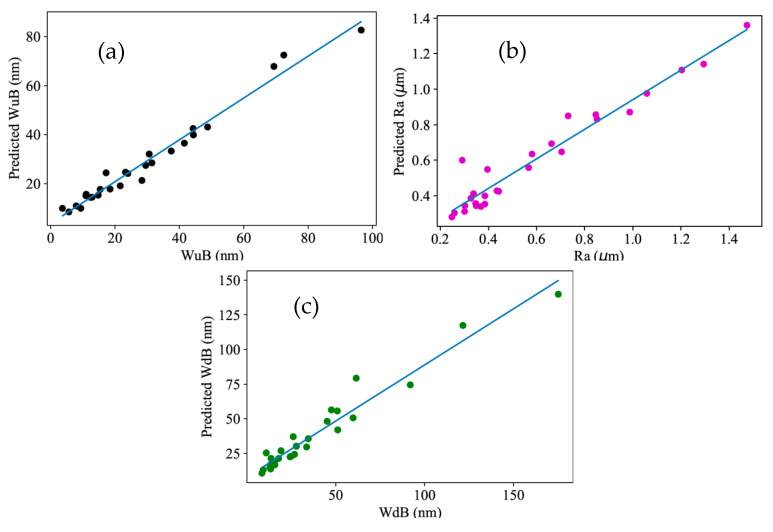
The relationship between the experiment results and RFR predicted results for the responses (**a**) Ra; (**b**) WdB; (**c**) WuB.

To improve upon the prediction accuracy, a random forest regression (RFR) model is adopted due to its interpretability, robustness, and strong predictive performance in manufacturing contexts compared to the Support Vector Regression (SVR) and Artificial Neural Networks (ANNs). The variations in the actual versus RFR-predicted responses are shown in Figure 5. It can be seen that the prediction accuracy of the RFR model is significantly better with R^2^ values of 0.93, 0.93, and 0.96 for the responses *R*_a_, WdB, and WuB, respectively. The RFR model’s ability to capture nonlinearity, handle feature interactions, robustness to outliers, ability to handle non-numerical features, and feature selection mechanisms collectively contribute to its superior performance. This is specifically true during the modelling of complex process mechanisms like micro-milling. More in-depth error analyses, such as RMSE and MAE, could be further explored if needed.

### 4.3. Multi-Objective Optimization Using GRA-TOPSIS

**Figure 6:**
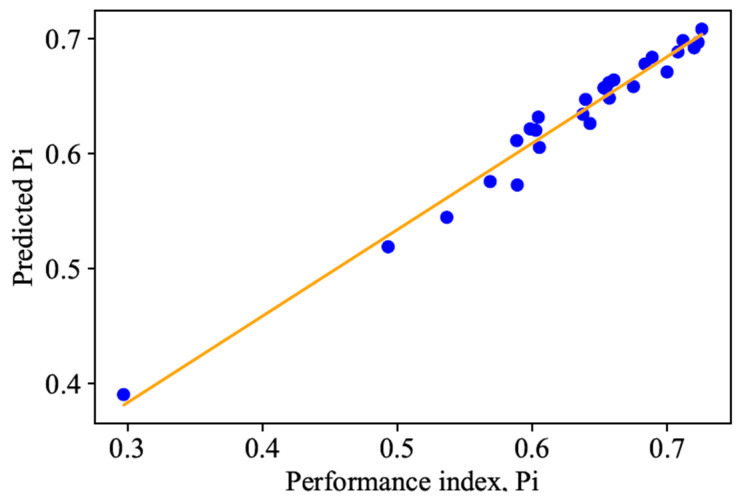
Comparison of actual vs. RFR predicted Pi (GRA TOPSIS).

The GRA-TOPSIS ranking matrix is computed next. The weighted normalized decision matrix is computed using Equations (1)–(3) and is given in Table 8. An unbiased weighting scheme is followed in this study. Table 9 lists the “best” and “worst” grey relational coefficients (GRC+ and GRC−), “best” and “worst” grey relational grades (GRG+ and GRG−), and performance indicator (Pi) for all responses as per Equations (4)–(10). As shown in Figure 6, the value of Pi indicates the overall performance of the process with respect to the responses under consideration. The alternatives are ranked based on the decreasing order of Pi with Pi = 0.725 fetching the highest rank, corresponding to the parameter combination A_3_B_1_C_2_ (spindle speed = 25,000 rpm; depth of cut = 0.05 mm; feed per tooth = 0.3 µm/tooth). The overall performance improvement in this parameter combination in terms of superior surface finish and burrs with respect to lower-ranked settings is verified in the subsequent section.

The method only ranks the parameter combinations within the dataset considered. Evaluating the performance of any parameter combinations outside the considered dataset can be made possible by modelling the performance indicator Pi using RFR. An R^2^ value of 0.947 is obtained, and the results are plotted as shown in Table 8.

**Figure 7:**
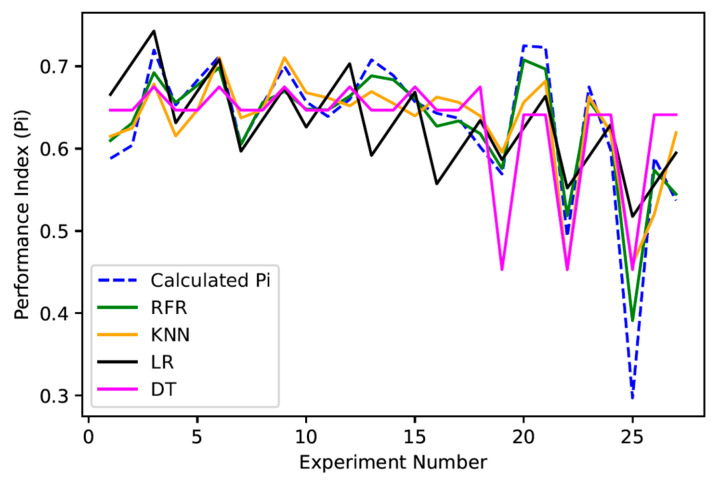
Performance index prediction result comparison.

The performance of the RFR is compared against other ML models next. For this, four ML models are selected, namely, linear regression, K nearest neighbours (KNNs), and decision tree (DT) regression. It can be observed as listed in Table 10 that RFR outperformed other models significantly with respect to overall prediction accuracy for the current dataset. This can also be further referred to in Figure 7 where the Pi predictions from multiple models are plotted with respect to the experiment number. The trained RFR model can be utilized to have a relative performance measure of any parameter settings, from the basic understanding that the settings with larger Pi predictions would perform better than the ones with lower Pi predictions.

**Figure 8:**
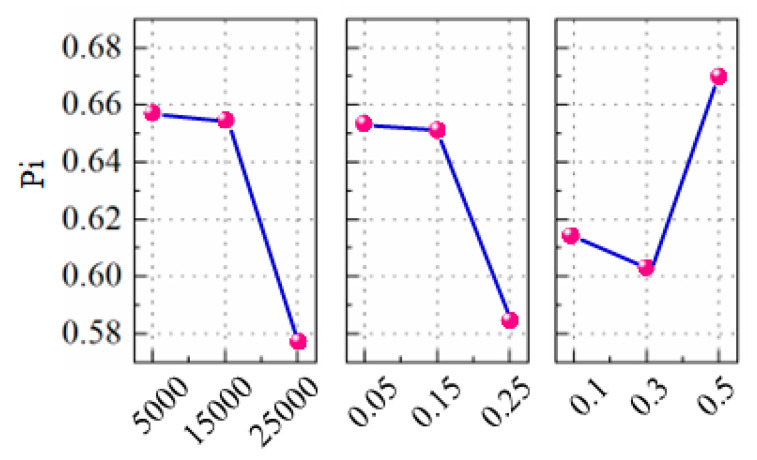
Main effect plot of ANOVA for Pi.

In practical cases, analyzing the overall performance as a single response is more beneficial than investigating individual performances. In the present case, the performance index in GRA-TOPSIS can be considered as a single parameter that represents the overall performance of the micro-milling process. As seen earlier, a higher value for the performance index indicates an overall better performance in terms of *R*_a_, WuB, and WdB. The response table for the mean of the closeness coefficient is shown in Table 11. From the mean response table and the main effect plot of Pi in Figure 8, it is clear that spindle speed has the maximum effect on the overall performance followed by the depth of cut and, finally, feed/tooth. Additionally, to study the relative effect of process parameters on the performance index (P_i_), ANOVA is performed, the details of which are shown in Table 12. From the ANOVA results, it is worth noting that the individual contribution of spindle speed is lower compared to the other two parameters, but it has sizable interaction effects.

For all of the results, the above analysis clarifies that the combination of GRA-TOPSIS and random forest regression (RFR) can provide a powerful and effective tool for solving complex manufacturing problems. GRA can help in selecting and ranking the most relevant features towards the overall performance improvement in the micro-milling process. Its capabilities are further enhanced by its integration with the TOPSIS approach. Moreover, the RFR model can handle nonlinear and complex relationships between input variables and output variables. The presented model can achieve high prediction accuracy with low error rates for optimization and prediction cases, as demonstrated earlier. Overall, the GRA-RFR hybrid model offers a promising approach for data analysis and decision-making in smart manufacturing, especially for complex precision manufacturing applications.

### 4.4. Confirmation of the Optimzied Paramters in Experments

Based on the above discussion in Section 4.3, the parameter settings of A3B1C2 were found optimal from the main effect plot of the closeness coefficient using the GRA-TOPSIS method. To validate the effectiveness of this finding, the corresponding experimental results (i.e., surface roughness and milling burrs at both up- and down-milling sides) generated by A3B1C2 were presented here and compared with the results generated by the optimal parameter settings of A1B3C2 and A3B3C1, which had been identified as the middle (i.e., 13th) and worse (i.e., 25th) combinations by the GRA-TOPSIS ranking in Table 9.

**Figure 9:**
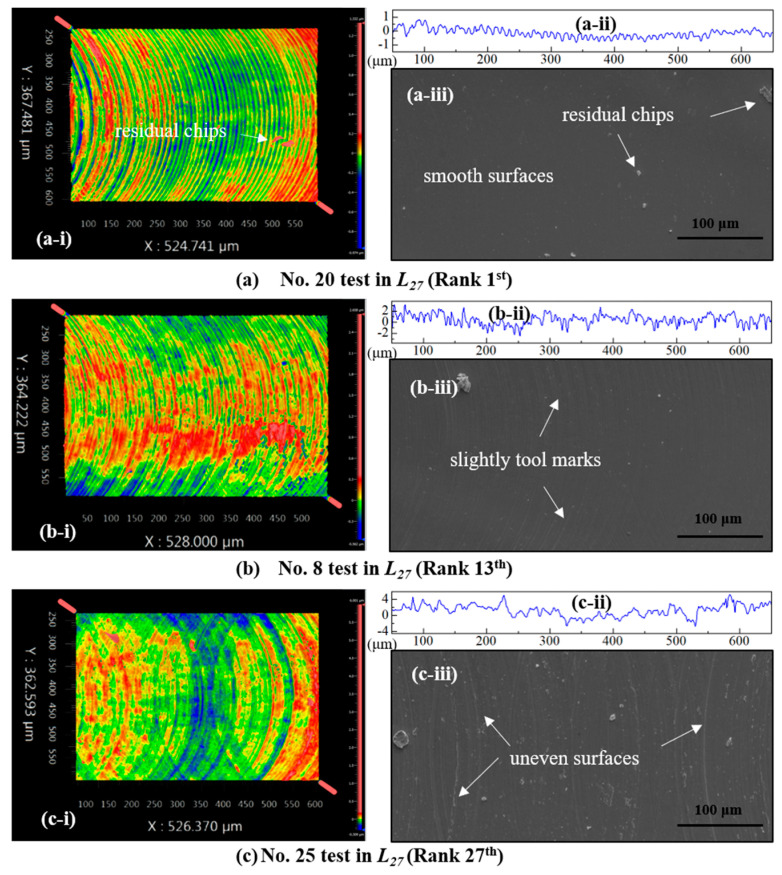
Surface morphologies, surface contours, and SEM image of the machined micro-grooves in the test No. 20 (**a**), No. 8 (**b**), and No. 25 (**c**). Based on the proposed GRA-TOPISS method, the rank of the No. 20 test is the highest (rank 1st), while that of No. 25 is the lowest (rank 27th), and the rank of No. 8 is in the middle (rank 13th).

Figure 9a–c show the measured surface morphologies, surface contours, and the SEM images of the micro-milled grooves with the parameter settings of A3B1C2 (rank 1st), A1B3C2 (rank 13th), and A3B3C1 (rank 25th), respectively. One can see that, at the optimal cutting conditions (A3B1C2), there are clear and trim residual tool marks on the generated groove surfaces, as shown in Figure 9a, although a few residual chips have adhered on the machined surfaces; on the other hand, in the other cutting conditions, the tool marks are prone to severe fluctuations, as shown in Figure 9b,c, especially in the case of parameter settings ranked 25th. In addition, the groove surface in Figure 9a is much smoother than those in the other two cases. This scenario could be proved by the measured surface roughness; in other words, *R*_a_ = 0.248 μm in Figure 9a is only around one-third or one-quarter of those in Figure 9b,c.

**Figure 10:**
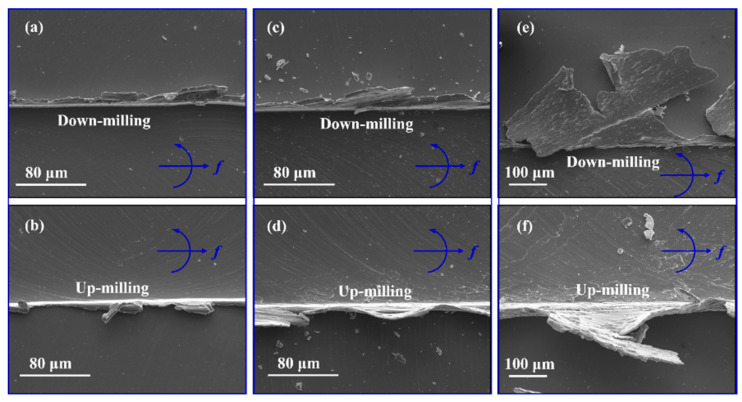
The burr morphologies generated in the down- and up-milling modes during micro-groove milling: (**a**,**b**) N = 25,000 rpm, a_p_ = 0.05 mm, f_z_ = 0.3 µm/tooth; (**c**,**d**) N = 5000 rpm, a_p_ = 0.25 mm, f_z_ = 0.3 µm/tooth; (**e**,**f**) N = 25,000 rpm, a_p_ = 0.25 mm, f_z_ = 0.1 µm/tooth.

Figure 10 presents the observed SEM images of the burr morphologies generated in down- and up-milling modes under the optimal parameter settings and comparison groups. One can see that the burrs generated by the down-milling process are always large than that generated by the up-milling process. As mentioned above, in the down-milling mode, the cutting edge moves away from the workpiece material after the UCT reduces from its maximum (*f*z) to zero. This means that the UCT might be below the critical value of chip formations at a certain moment in the down-milling mode. Once it happens, the cutting edge would stack the material to the groove edges instead of removing the materials through chip formation, which has been identified as a ploughing effect [36,39,40], resulting in the formation of burrs. Moreover, it is worth noting that the micro-milled grooves with the optimal parameter settings in Figure 10a witness the smallest burrs in size when compared with those in Figure 10b,c. In particular, as shown in Figure 10c, the width of the burrs generated by the A3B3C1 (rank 25th) is up to around 200 μm, indicating quite poor and unacceptable machined surface quality. This scenario should be attributed to the severe ploughing effect [41,42] caused by the small depth of cut and feed rate per tooth. Moreover, there are lots of residual marks left on the machined surfaces by the secondary cutting edges via the plough effect, resulting in poor surface finish. In summary, through analyzing the machined surface morphologies, the optimal parameter settings by GRA-TOSIS are proven to produce the optimal surface quality and should be recommended in the practical machining of Ti6-Al4-V alloys for the industrial manufacturing of miniaturized products. Moreover, the proposed approach will be used, in the ongoing work, to predict the machining quality of new parameter combinations.

## 5. Conclusions

To enhance the machining quality of difficult-to-cut titanium alloys, this study proposes a hybrid ranking algorithm to optimize the burr formation in the micro-milling process of Ti-6Al-4V alloys, which integrates the random forest regression (RFR) model for the first time with Grey Relational Analysis (GRA) and Technique for Order Preference by Similarity to Ideal Solution (TOPSIS) methods. Based on the proposed hybrid GRA-TOPSIS-RFR approach, the micro-milling process of the Ti-6Al-4V alloy was modelled and optimized, and the validation experimental results agree well with the optimized outcomes. The main findings can be summarized below:

(1) The hybrid GRA-TOPSIS-RFR optimization algorithm proposed in this work can leverage the strengths of RFR models to handle complex, nonlinear relationships between micro-milling parameters and the optimization performance index. RFR being more accurate, robust, and explainable can be integrated effectively with GRA-TOPSIS to model and optimize challenging manufacturing problems.
(2) Linear regression and RFR models were built for predicting the surface roughness (*R*_a_), burr widths on the down-milling side (WdB), and burr widths on the up-milling side (WuB). For the given dataset, the RFR model outperformed the linear regression models with R^2^ values of 0.93, 0.93, and 0.96 against 0.7009, 0.5591, and 0.7164 for *R*_a_, WdB, and WuB, respectively.
(3) The surface roughness has a positive correlation with the spindle speed and depth of cut, while *R*_a_ was found to increase with a small feed rate due to the ploughing effect. Both burr widths (i.e., WuB and WdB) were found to first decrease and then increase with the rise in the spindle speed and depth of cut, while burr widths always showed a decreasing trend with the increase in feed rate.
(4) The depth of cut has the largest influence on the surface roughness, while the feed rate per tooth plays the most important role in burr formation in both down- and up-milling processes.
(5) Based on the GRA-TOPSIS-RFR approach, the optimal parameter combination for micro-milling Ti-6Al-4V was given as spindle speed *N* = 25,000 RPM, depth of cut *a*_p_ = 0.05 mm, and feed rate per tooth *f*_z_ = 0.3 μm/tooth. Overall, the GRA-RFR hybrid model offers a promising approach for data analysis and decision-making in smart manufacturing, especially for complex precision manufacturing applications.

The proposed hybrid optimization technique has shown great potential for optimizing the micro-milling process, which is an otherwise challenging task due to the complex machining mechanism and higher-order parameter interactions. Though the presented approach is very promising in its current state towards overall performance enhancement, it has the potential to be further improved in future. Some future research directions include testing the robustness of the proposed approach with different manufacturing processes, quantifying and addressing model uncertainties, and extending the approach for real-time optimization applications.

## Figures and Tables

**Table 1:** Chemical composition of Ti-6Al-4V.

**Chemical** **Elements**	V	Al	Sn	Zr	Mo	C	Si	Cr	Fe	Cu	Nb	Ti
**Weight** **(%)**	4.22	5.48	0.0625	0.0025	0.005	0.369	0.022	0.0099	0.112	<0.02	0.0386	90

**Table 2:** Mechanical properties of Ti-6Al-4V.

Parameter	Value
Tensile strength (MPa)	950
Elastic modulus (GPa)	114
Density (g/cm^3^)	4.42
Vickers hardness (kgf/mm^2^)	330

**Table 3:** The parameters used in micro-milling tests.

Symbol	Factors	Level		
		1	2	3
A	Spindle speed N (RPM)	5000	15,000	25,000
B	Depth of cut *a*_p_ (mm)	0.05	0.15	0.25
C	Feed per tooth *f*_z_ (μm/tooth)	0.1	0.3	0.5

**Table 4:** The L_27_ array and response results.

Exp.	Group	Factors			Response Results		
		A	B	C	R_a_ (μm)	WdB (μm)	WuB (μm)
1	A1B1C1	5000	0.05	0.1	0.349	92.05	44.25
2	A1B1C2	5000	0.05	0.3	0.385	59.83	48.76
3	A1B1C3	5000	0.05	0.5	0.338	13.56	11.03
4	A1B2C1	5000	0.15	0.1	0.347	45.23	31.45
5	A1B2C2	5000	0.15	0.3	0.302	33.68	23.95
6	A1B2C3	5000	0.15	0.5	0.581	8.53	3.65
7	A1B3C1	5000	0.25	0.1	0.662	50.95	29.53
8	A1B3C2	5000	0.25	0.3	0.704	13.68	23.24
9	A1B3C3	5000	0.25	0.5	0.291	13.45	28.35
10	A2B1C1	15,000	0.05	0.1	0.259	47.62	37.46
11	A2B1C2	15,000	0.05	0.3	0.301	51.18	41.53
12	A2B1C3	15,000	0.05	0.5	0.567	24.35	21.62
13	A2B2C1	15,000	0.15	0.1	0.385	19.23	10.95
14	A2B2C2	15,000	0.15	0.3	0.443	17.92	18.45
15	A2B2C3	15,000	0.15	0.5	0.987	12.98	5.67
16	A2B3C1	15,000	0.25	0.1	0.846	26.13	12.45
17	A2B3C2	15,000	0.25	0.3	0.851	24.89	15.39
18	A2B3C3	15,000	0.25	0.5	1.203	15.56	14.78
19	A3B1C1	25,000	0.05	0.1	0.369	61.58	69.35
20	A3B1C2	25,000	0.05	0.3	0.248	10.96	17.22
21	A3B1C3	25,000	0.05	0.5	0.396	9.24	7.95
22	A3B2C1	25,000	0.15	0.1	0.435	121.65	72.42
23	A3B2C2	25,000	0.15	0.3	0.327	27.89	30.61
24	A3B2C3	25,000	0.15	0.5	1.294	15.8	9.35
25	A3B3C1	25,000	0.25	0.1	1.058	175.32	96.48
26	A3B3C2	25,000	0.25	0.3	0.731	34.58	44.35
27	A3B3C3	25,000	0.25	0.5	1.474	26.88	12.86

**Table 5:** ANOVA results for *R_a_.*

Sour.	Fre.	Seq SS	Adj SS	Adj MS	F	*p*	Cont. (%)
A	2	0.3488	0.3488	0.1744	8.75	0.010	11.1
B	2	1.1924	1.1924	0.5962	29.92	0.000	37.8
C	2	0.5221	0.5221	0.2610	13.10	0.003	16.6
A∗B	4	0.2466	0.2466	0.0616	3.09	0.082	7.8
A∗C	4	0.4200	0.4200	0.1050	5.27	0.022	13.3
B∗C	4	0.2594	0.2594	0.0648	3.25	0.073	8.24
RE	8	0.1594	0.1594	0.0199			5.1
Total	26	3.1486					

**Table 6:** ANOVA results for WdB.

Sour.	Fre.	Seq SS	Adj SS	Adj MS	F	*p*	Cont. (%)
A	2	3379.5	3379.5	1689.7	4.52	0.049	9.0
B	2	401.5	401.5	200.7	0.54	0.604	1.1
C	2	14,843.4	14,843.4	7421.7	19.83	0.001	39.5
A∗B	4	6122.6	6122.6	1530.7	4.09	0.043	16.3
A∗C	4	8944.5	8944.5	2236.1	5.98	0.016	23.8
B∗C	4	930.3	930.3	232.6	0.62	0.660	2.5
RE	8	2993.5	2993.5	374.2			8.0
Total	26	37,615.2					

**Table 7:** ANOVA results for WuB.

Sour.	Fre.	Seq SS	Adj SS	Adj MS	F	*p*	Cont. (%)
A	2	88.73	88.73	44.37	4.72	0.044	7.2
B	2	106.76	106.76	53.38	5.68	0.029	8.6
C	2	537.53	537.53	268.76	28.58	0.001	43.4
A∗B	4	157.66	157.66	39.41	4.19	0.040	12.7
A∗C	4	187.91	187.91	46.98	4.99	0.026	15.2
B∗C	4	83.95	83.95	20.99	2.23	0.155	6.8
RE	8	75.24	75.24	9.41			1
Total	26	1237.8					

**Table 8:** Normalized decision matrix.

S. No.	N-*R*_a_	N-WdB	N-WuB	W-N-*R*_a_	W-N-WdB	W-N-WuB	GRC+ *R*_a_
1	0.10	0.33	0.23	0.03	0.11	0.08	0.86
2	0.11	0.21	0.26	0.04	0.07	0.09	0.82
3	0.09	0.05	0.06	0.03	0.02	0.02	0.87
4	0.10	0.16	0.17	0.03	0.05	0.06	0.86
5	0.08	0.12	0.13	0.03	0.04	0.04	0.92
6	0.16	0.03	0.02	0.05	0.01	0.01	0.65
7	0.19	0.18	0.16	0.06	0.06	0.05	0.60
8	0.20	0.05	0.12	0.07	0.02	0.04	0.57
9	0.08	0.05	0.15	0.03	0.02	0.05	0.93
10	0.07	0.17	0.20	0.02	0.06	0.07	0.98
11	0.08	0.18	0.22	0.03	0.06	0.07	0.92
12	0.16	0.09	0.11	0.05	0.03	0.04	0.66
13	0.11	0.07	0.06	0.04	0.02	0.02	0.82
14	0.12	0.06	0.10	0.04	0.02	0.03	0.76
15	0.28	0.05	0.03	0.09	0.02	0.01	0.45
16	0.24	0.09	0.07	0.08	0.03	0.02	0.51
17	0.24	0.09	0.08	0.08	0.03	0.03	0.50
18	0.34	0.06	0.08	0.11	0.02	0.03	0.39
19	0.10	0.22	0.37	0.03	0.07	0.12	0.84
20	0.07	0.04	0.09	0.02	0.01	0.03	1.00
21	0.11	0.03	0.04	0.04	0.01	0.01	0.81
22	0.12	0.43	0.38	0.04	0.14	0.13	0.77
23	0.09	0.10	0.16	0.03	0.03	0.05	0.89
24	0.36	0.06	0.05	0.12	0.02	0.02	0.37
25	0.30	0.62	0.51	0.10	0.21	0.17	0.43
26	0.20	0.12	0.23	0.07	0.04	0.08	0.56
27	0.41	0.10	0.07	0.14	0.03	0.02	0.33

**Table 9:** GRA-TOPSIS-based ranking of alternatives.

S. No.	GRC+ *R*_a_	GRC+ WdB	GRC+ WdC	GRC− *R*_a_	GRC− WdB	GRC− WdC	GRG+	GRG−	Pi	Rank
1	0.86	0.50	0.53	0.35	0.50	0.47	0.63	0.44	0.588	23
2	0.82	0.62	0.51	0.36	0.42	0.49	0.65	0.42	0.604	19
3	0.87	0.94	0.86	0.35	0.34	0.35	0.89	0.35	0.720	3
4	0.86	0.69	0.63	0.35	0.39	0.42	0.73	0.39	0.653	14
5	0.92	0.77	0.70	0.34	0.37	0.39	0.79	0.37	0.683	8
6	0.65	1.00	1.00	0.41	0.33	0.33	0.88	0.36	0.712	4
7	0.60	0.66	0.64	0.43	0.40	0.41	0.63	0.41	0.605	18
8	0.57	0.94	0.70	0.44	0.34	0.39	0.74	0.39	0.654	13
9	0.93	0.94	0.65	0.34	0.34	0.41	0.84	0.36	0.700	6
10	0.98	0.68	0.58	0.34	0.40	0.44	0.75	0.39	0.657	11
11	0.92	0.66	0.55	0.34	0.40	0.46	0.71	0.40	0.639	16
12	0.66	0.84	0.72	0.40	0.36	0.38	0.74	0.38	0.660	10
13	0.82	0.89	0.86	0.36	0.35	0.35	0.86	0.35	0.708	5
14	0.76	0.90	0.76	0.37	0.35	0.37	0.81	0.36	0.689	7
15	0.45	0.95	0.96	0.56	0.34	0.34	0.79	0.41	0.657	12
16	0.51	0.83	0.84	0.49	0.36	0.36	0.72	0.40	0.643	15
17	0.50	0.84	0.80	0.50	0.36	0.36	0.71	0.41	0.637	17
18	0.39	0.92	0.81	0.69	0.34	0.36	0.71	0.47	0.602	20
19	0.84	0.61	0.41	0.36	0.42	0.63	0.62	0.47	0.569	24
20	1.00	0.97	0.77	0.33	0.34	0.37	0.92	0.35	0.725	1
21	0.81	0.99	0.92	0.36	0.33	0.34	0.90	0.35	0.723	2
22	0.77	0.42	0.40	0.37	0.61	0.66	0.53	0.55	0.493	26
23	0.89	0.81	0.63	0.35	0.36	0.41	0.78	0.37	0.675	9
24	0.37	0.92	0.89	0.77	0.34	0.35	0.73	0.49	0.598	21
25	0.43	0.33	0.33	0.60	1.00	1.00	0.37	0.87	0.297	27
26	0.56	0.76	0.53	0.45	0.37	0.47	0.62	0.43	0.589	22
27	0.33	0.82	0.83	1.00	0.36	0.36	0.66	0.57	0.537	25

**Table 10:** Comparisons of the performance of linear regression, DT, SVR, and KNN models during the prediction of the GRA-TOPSIS performance index.

S. No.	ML Models	Prediction Accuracy (R^2^)
1	Linear regression (LR)	0.5016
2	Decision tree (DT)	0.7236
4	K nearest neighbours (KNNs)	0.866
5	Random forest regression (RFR)	0.947

**Table 11:** Response table for the mean of closeness coefficient.

Parameters	A	B	C
Level 1	0.6577	0.6539	0.6178
Level 2	0.6547	0.6520	0.6030
Level 3	0.5784	0.5849	0.6700
Delta	0.0792	0.0690	0.0670
Rank	1	2	3

**Table 12:** ANOVA results for Pi.

Sour.	Fre.	Seq SS	Adj SS	Adj MS	F	*p*	Cont. (%)
A	2	88.73	88.73	44.37	4.72	0.044	7.2
B	2	106.76	106.76	53.38	5.68	0.029	8.6
C	2	537.53	537.53	268.76	28.58	0.001	43.4
A∗B	4	157.66	157.66	39.41	4.19	0.040	12.7
A∗C	4	187.91	187.91	46.98	4.99	0.026	15.2
B∗C	4	83.95	83.95	20.99	2.23	0.155	6.8
RE	8	75.24	75.24	9.41			1
Total	26	1237.8					

## Data Availability

Dataset available on request from the authors.
